# The Ethnobotanical, Phytochemical and Mineral Analyses of *Phragmanthera Incana* (Klotzsch), A Species of Mistletoe Growing on Three Plant Hosts in South-Western Nigeria

**Published:** 2013-03

**Authors:** O. T. Ogunmefun, T. R. Fasola, A. B. Saba, O. A. Oridupa

**Affiliations:** 1 *epartment of Biological Sciences, College of Sciences, Afe Babalola University, Ado-Ekiti, Nigeria*; 2 *Department of Botany, Faculty of Science, University of Ibadan, Ibadan, Nigeria*; 3 *Department of Physiology, Biochemistry and Pharmacology, Faculty of Veterinary Medicine, University of Ibadan, Ibadan, Nigeria*

**Keywords:** *irvingia gabonensis*, *kola nitida*, mineral analysis, phragmanthera incana, *theobroma cacao*, phytochemical analysis

## Abstract

Mistletoe is collected wildly on various plants and *Phragmanthera incana* is noted to grow on different plant hosts. This study was designed to carry out the ethnobotanical survey, phytochemical and mineral analyses of *Phragmanthera incana*, a species of mistletoe growing on three plant hosts namely Cocoa (*Theobroma cacao*), Kolanut (*Cola nitida*) and Bush mango (*Irvingia gabonensis*). Mistletoe samples were identified at the Forestry Research Institute of Nigeria Herbarium. *Phragmanthera incana* was screened for its phytochemical constituents and mineral cations along its hosts following standard methods and to confirm if the mistletoe species is host specific. The powdered samples of the mistletoe species (*Phragmanthera incana*) was used for both the phytochemical screening and the cation mineral analysis. The uses and the harvesting methods of mistletoe were also reviewed extensively in this paper.

## INTRODUCTION

Mistletoe is a general term for woody shoot parasites in several plant families, especially in Loranthaceae and Viscaceae families (16). The common European mistletoe grows on various trees, usually apples and junipers. It is an evergreen plant with small, greenish flowers and berries. A similar mistletoe, American mistletoe, found in the United States, grows on deciduous trees, particularly red marple elm, from eastern Texas to Florida and northward to Missouri and New Jersey (18). The leafless flowering dwarf mistletoes depend entirely on the host tree for nourishment. These scrubs are lethal parasites of conifers, such as pine, spruce, fir and hemlock. The plant leaves and berries contain toxic chemicals that can be poisonous and the plant should be kept out of reach of young children who may be tempted to eat the berries (18).

The common mistletoe figured significantly in the folklore and religions of pre – Christian Europe. Reputedly endowed with magical powers, it was used as a remedy for evil; it is used as a Christmas and New Year’s decoration, and kissing under a branch of mistletoe is still customary (18). The common European mistletoe is classified scientifically as *Viscum album* L., the American mistletoe as *Phoradendron flavescens* (Pursh Nutt.) and the dwarf mistletoe as *Arceuthobium pusillum* (18). Many ornamental plants, such as oleander, lily of the valley and mistletoe are poisonous (14).

Over the next one thousand years, the observance of Christmas followed the expansion of Chistianity into the rest of Europe and into Egypt. Along the way, Christian beliefs combined with existing pagan feasts and winter rituals to create many long-standing traditions of Christmas celebrations. For example, ancient Europeans believed that the mistletoe plant held magical powers to bestow life and fertility, to bring peace, and to protect against disease. Northern Europeans associated the plant with Norse goddess of love, Freya, and developed the custom of kissing underneath mistletoe branches. Christians incorporated this custom into their Christmas celebrations, and kissing under a mistletoe branch eventually became a part of secular Christmas tradition (19).

Most genera of African mistletoes belong to the family Loranthaceae (17). In West Africa, mistletoes are found on many indigenous trees and a number of tree crops of economic importance (3), including sheabutter, neem, citrus (4), cocoa (16) and rubber (9, 10).

In the Southwestern Nigeria, mistletoe is commonly found growing especially on tree crops like cocoa, kola, coffee, bush mango etc known botanically as *Theobroma cacao* L., *Cola nitida* (Vent.) Schott & Endl. or *Cola acuminata (P. Beauv.)* Schott & Endl., *Coffea Arabica* L. and *Irvingia gabonensis* (Aubrey-Lecomte ex O. Rorke) Baill. respectively. Mistletoe can also be found growing on citrus plants like orange (*Citrus sp*.), guava (*Psidium guajava* L.) etc.

Mistletoe is especially interesting botanically because it is a partial parasite (a “hemiparasite”) (18). As a parasitic plant, it grows on the branches of trunk of trees and actually sends out haustoria that penetrate into the tree and take up nutrients (20). Mistletoe is also capable of growing on its own; like other plants as it can produce its own food by photosynthesis.

Mistletoe, however, is more commonly found growing as a parasitic plant. The mistletoe that is commonly used as a Christmas decoration [*Phoradendron flavescens* (Pursh Nutt.)] is native to North America and grows as a parasite on trees from New Jersey to Florida. The other type of mistletoe, *Viscum album* L., is of European origin. The Greeks and earlier peoples thought that it had mystical powers and down through the centuries it became associated with many folklore customs. The European mistletoe is a green shrub with small, yellow flowers and white, sticky berries which are considered poisonous. It is commonly seen on apple but only rarely on oak trees.

The common name of the plant is derived from the ancient belief that mistletoe was propagated from bird droppings. The belief was related to the then-accepted principle that life could spring spontaneously from dung. It was observed in ancient times that mistletoe would often appear on a branch or twig where birds had left droppings. “Mistletoe” is the Anglo-saxon word for “dung” and “tan” is the word for “twig”. So, mistletoe means “dung-on-a-twig” (20).

### Botanical Description of *Phragmanthera incana* FHI 108925

*Phragmanthera incana* is a woody parasitic shrub, stems to 2m long; of secondary jungle and bush savanna areas; from Sierra Leone to West Cameroons and Fernando Po Island (gulf of Guinea that forms part of Equatorial Guinea), and extending across the Congo basin to Zaire and Angola. The plant is very variable in form, common and widely distributed (5). Young parts and perianth more or less densely covered with brown hairs; berries red. The plant is very variable in the shape and size of the flowers and leaves. It’s found on *Alchornea castaneifolia, Anacardium occidentale, Aleurites molluccana, Bauhinia monandra, Bombax sessile* (8).

## USES OF MISTLETOE

Mistletoe is used mainly in Europe as a treatment for cancer (15). While American mistletoe is toxic, European mistletoe is considered to have medicinal properties till today. The Drug Digests states that “for several diseases, European mistletoe has been used to treat a wide variety of physical and mental conditions. Currently, it is best known as an additional therapy with other drugs and or radiation for treating cancer”. Some HIV/AIDS Organizations (NGO’s) also claim that it can help restore immune systems (11).

Away from superstitious beliefs, mistletoe has been used in medicine to prove much of its older frame as “all healer”. The white-berried mistletoe (*Viscum album*) has been documented as a traditional treatment for diabetes and high blood pressure. Mistletoe extracts represent the most unorthodox oncology therapy in Germany (15). Ethnobotanical surveys carried out in Palestine showed the use of this plant (*Viscum album*) to treat skin diseases and prostate cancer (13). In Nigeria, the Hausa and Fulani tribes of Northern Nigeria use mistletoe in the treatment of cancers and inflammations. (1).

The African mistletoe, *Loranthus bengwensis* L. (Loranthaceae), has been widely used in Nigeria folk medicine to treat *Diabetes mellitus* (12). Another type of African mistletoe *Tapinanthus dodoneifolius* revealed a wide spectrum of antimicrobial activities against certain multiple drug resistant bacteria and fungal isolates of farm animals. The inhibition of *Bacillus sp. Escherichia coli, Salmonella sp., Proteus sp., Pseudomonas sp., Agrobacterium tumefaciens*, bacterial sp., known to be associated with either crown gall or gastrointestinal tract and wound infections by this plant extract gives clue to its ethnomedicinal usage (7). Mistletoe leaves have been reported for treating cholera, nerves and heart problems (11).

Mistletoe is also useful for the treatment of insomnia as it relaxes muscles, calms the nerves, eases palpitation, migraine, nervousness and pains. It has also been observed to slow down the attack of epilepsy and for treating fibroids. Mistletoe is used to treat arthritis, rheumatism and gout as it increases the production of urine and the elimination of toxic waste from the system (2). Mistletoe leaves contain choline and acetylcholine. Though these compounds act directly on the autonomic nervous system, the berries contain alkaloids and toxic substances and should not be ingested (5).

## HARVESTING METHODS

### Use of Go-to-hell

Used for harvesting mistletoe from trees of high canopy. A very long stick is used to remove mistletoe from trees that are very high and not within reach (5).

### Use of Sickles

This method is suitable for harvesting mistletoe from young trees of very low canopy; sickles have curved ends which make it easier to remove mistletoe from trees that are not too high (5).

### Use of Cutting Knives/Cutlasses

For harvesting mistletoe from trees of very low canopy. Knives or cutlasses are used to cut down mistletoe from trees that are not high since it’s within reach (5).

### Prunning

In this method, a heavily infested branch of a tree is completely cut off (prunned) from the plant with the mistletoe later removed (5).

### Use of Climbing Equipment

This method is used to remove mistletoe from trees that are already bearing fruits to avoid destruction of the fruits. The climbing equipment is used to get to the tree canopies and the mistletoe is carefully removed using cutting knives (5).

This study on *Phragmanthera incana*, a mistletoe species found growing on three major tree crops in the South-western states of Nigeria was aroused to confirm if the mistletoe species is host specific by carrying out its phytochemical and mineral cation analyses along with its hosts and to confirm its ethnomedicinal uses and harvesting methods as found in different literatures.

## MATERIALS AND METHODS

### Plant Sample Collection

The methods used in this work involved collection of the mistletoe (*Phragmanthera incana*) growing on Cocoa (*Theobroma cacao*), Kolanut (*Cola nitida*) and Bush mango (*Irvingia gabonensis*) along with leaves and stem bark of the respective hosts. The collection was done in an area called Alesan Obolode, Owo, Ondo State, Nigeria in July, 2010. Larger quantities were collected late January, 2011 after which identification and authentication was done at the Forestry Research Institute of Nigeria (FRIN) herbarium. A voucher specimen of *Phragmanthera incana* with Forestry Herbarium Index (FHI) 108925 was submitted at the Botany Department herbarium of the University of Ibadan (5).

Mistletoe, the leaves along with stem bark of host plants were air dried for about a week, after which they were milled separately at the Wood Extraction unit of the Chemistry Department, University of Ibadan. The milled samples of the mistletoe species on both cocoa and kolanut trees were extracted with methanol using cold extraction method (cold maceration) and were concentrated using rotary evaporator. The concentrated extracts were further dried on water bath at a very low temperature of about 40°C at the Department of Pharmacognosy laboratory to eliminate all solvents.

### Ethnobotanical Survey

A survey was carried out around the South-western states of Nigeria using questionnaire to interview individuals who are knowledgeable on the subject matter. They include herb sellers and traditional medical practitioners about the usefulness of mistletoe in ethnomedicine and its harvesting methods.

### Phytochemical Analysis

The phytochemical screening of the powdered leaves and stem bark of the host plants along with the powdered mistletoe samples on the three host plants was done at the Pharmacognosy Department, Faculty of Pharmacy, University of Ibadan. Five major secondary metabolites screened were alkaloids, cardenolides, anthraquinones, saponins and tannins (Table 1).

**Table 1:** Phytochemical Analysis of Mistletoe and Host plant parts commonly used

	Alkaloids			Cardenolides		Anthraquinones	Saponins	Tannins
Plant Sample	Dragendoff’s	Meyer’s	Wagner’s	Keller- Killiani	Kedde	Chloroform/Ammonia	Frothing	Ferric chloride

TCL	+	+	+	+	-	-	+	+
TCM	+	+	+	+	±	-	+	+
TCS	+	-	-	-	+	-	-	-
IGL	+	+	+	+	-	-	-	+
IGM	+	-	-	+	+	-	+	-
IGS	-	-	-	+	+	+	+	+
KNL	+	-	-	+	±	-	-	-
KNM	+	-	-	+	+	-	+	-
KNS	-	-	-	+	+	-	+	-

+, Present; -, Absent; ±, Present in trace quantity/Doubtful; TCL, *Theobroma cacao* powdered leaf samples; TCM, Mistletoe harvested from *Theobroma cacao* tree; TCS, *Theobroma cacao* powdered stem bark; IGL, *Irvingia gabonensis* powdered leaf samples; IGM, Mistletoe harvested from *Irvingia gabonensis* tree; IGS, *Irvingia gabonensis* powdered stem bark; KNL, *Kola nitida powdered* leaf samples; KNM, Mistletoe harvested from *Kola nitida* tree; KNS, *Kola nitida* stem.

### Mineral Analysis

The mineral analysis of four major cations in the leaves and stem bark of host plants alongside mistletoe on the three host plants was also carried out on the powdered samples at the General Central Research Laboratory, University of Ibadan. The cations screened for were Calcium (Ca %), Magnesium (Mg %), Potassium (K %) and Sodium (mg Na/l) (Table 2).

**Table 2:** Major Mineral Cations of Powdered Plant Samples

S/No	Powdered Plant Samples	% Ca	% Mg	% K	mgNa/l

1	*Theobroma cacao* (leaf)	1.62	0.32	0.87	117
2	*Theobroma cacao* (mistletoe)	1.62	0.32	2.83	156
3	*Theobroma cacao* (stembark)	4.21	0.28	1.53	130
4	*Irvingia gabonensis* (leaf)	1.12	0.17	0.42	88
5	*Irvingia gabonensis *(mistletoe)	0.61	0.18	2.28	332
6	*Irvingia gabonensis *(stembark)	1.09	0.15	0.49	136
7	*Kola nitida* (leaf)	1.37	0.28	0.84	130
8	*Kola nitida* (mistletoe)	1.44	0.25	2.21	157
9	*Kola nitida* (stem bark)	1.92	0.15	0.95	199

In Table 2, all the elements tested were present in all the samples. The calcium present in Theobroma cacao stem bark had the highest value of 4.21%. This was followed by Potassium content of T. cacao mistletoe with 2.83%. The least value of 0.15% was observed in Magnesium of Kola nitida stem bark.

## RESULTS

### Ethnobotanical Report

The ethnobotanical survey in the South-western states of Nigeria showed that mistletoe is generally called “Afomo” in Yoruba language and used to treat many human and animal ailments like hypertension, diabetes, hepatitis, stroke, cancer and nervous disorders. Many of the people interviewed mentioned the use of long stick or sickle to harvest mistletoe from tall trees. Cutlasses can also be used to cut down branches that are heavily infested with mistletoe. The infusion is taken as tea over a period of one to two weeks and afterwards noticeable changes occur. It also helps in treating insomnia after which sound sleep results.

## DISCUSSION AND CONCLUSSION

The results gathered from the ethnobotanical survey on *Phragmanthera incana* stressed the reasons for its various uses in ethnomedicine. Different people who have used the mistletoe species testified to its effectiveness in treating insomnia, diabetes, hypertension, infertility e.t.c. Herb sellers in the South-western states of Nigeria who are professionals in their own fields also attested to these various uses claimed by the individuals.

The phytochemical analysis carried out in the course of this study showed that *Phragmanthera incana* contained alkaloids which is similar to the findings of Fasanu and Oyedapo (7). Though, only a little work has been done on the phytochemical and mineral compositions of this plant, it was found out that the leaves of the host plant and the mistletoe contained alkaloids and saponins with no anthraquinones in the case of cocoa while *Irvingia* and *Kola* leaves lacked saponins but the mistletoe on them contained saponins. Only *Irvingia* stem bark had anthraquinones. The leaves, mistletoe and stem bark of all the plants contained cardenolides. *Theobroma* *cacao* leaves and its mistletoe had tannins but the stem bark lacked tannins while there was no tannins found in *Kola* leaves, mistletoe and stem bark of the plant. *Irvingia* leaves and stem bark contained tannins but was absent in the mistletoe. *Theobroma* *cacao* leaves and its mistletoe had very similar chemical constituents (almost the same result for the five tests carried out on alkaloids, cardenolides, anthraquinones, saponins and tannins).

Phytochemicals help plants defend against environmental challenges and also provide humans with protection against various diseases as well. Hence it is not surprising while they are harvested for medicinal purposes (21).

**Figure 1:**
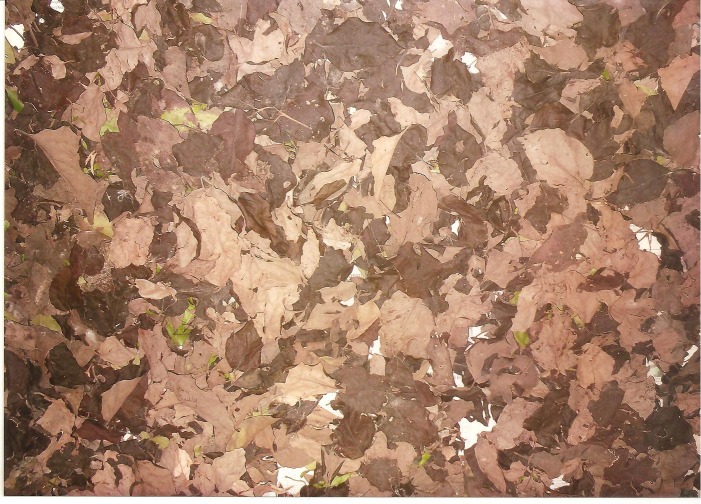
Air-dried sample of *Phragmanthera incana* (a mistletoe species) harvested from *Theobroma cacao* (Cocoa), Cola nitida (Kolanut) and *Irvingia gabonensis* (Bush Mango)

**Figure 2:**
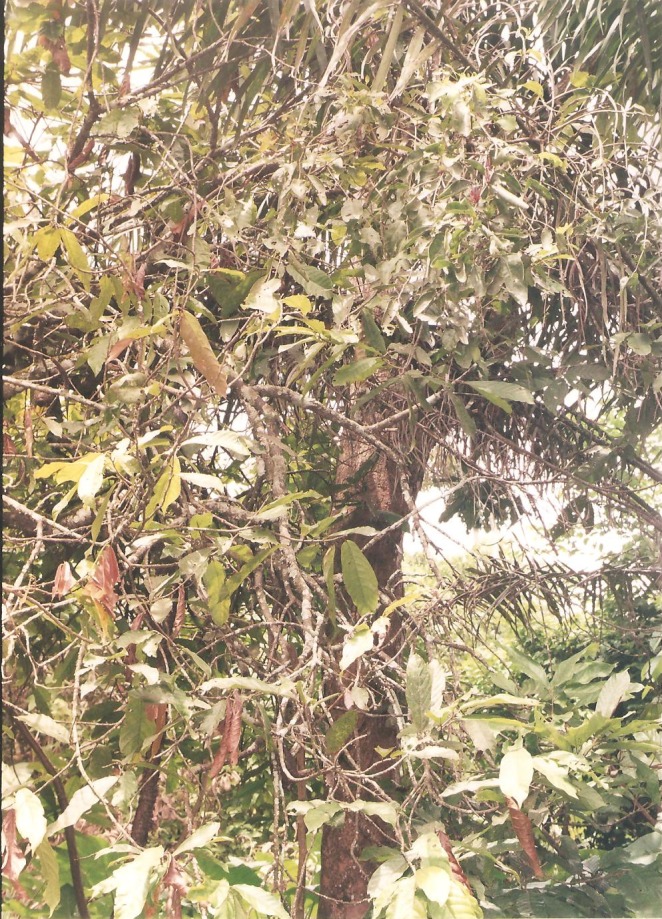
Mistletoe growing on *Theobroma cacao* (Cocoa) tree

**Figure 3:**
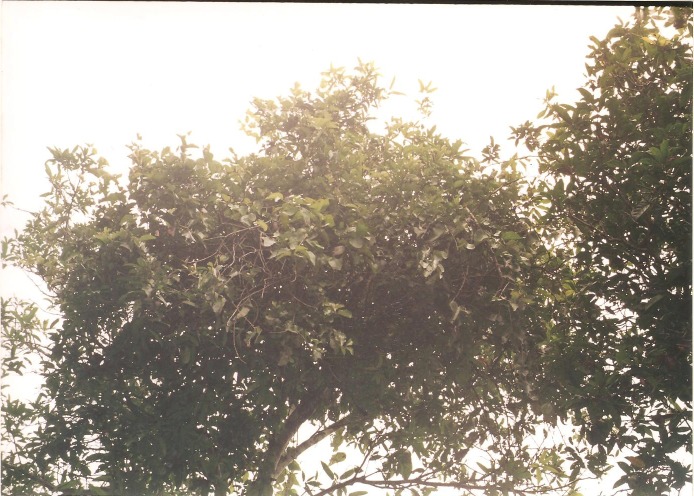
Mistletoe growing on *Cola nitida* (Kolanut) tree

Most similarities in chemical compositions are found between the leaves and the mistletoe in the three plants (cocoa, kolanut and bush mango) probably because both are leaves and the mistletoe most often attach themselves to the branches where leaves grow and not on the stem bark (Figure 1, Figure 2 and Figure 3).

The results of the mineral analysis of the major cations also show that the leaves, the stem bark of the host plants (cocoa, kolanut and bush mango) and the mistletoe growing on them contain major cations like potassium, magnesium, calcium and sodium in varying quantities. The same mistletoe species (*Phragmanthera incana*) growing on different plant hosts contain different quantities of mineral cations relative to the individual hosts. This study shows that *Phragmanthera incana* is not host specific having similarities with the chemical compositions of the leaves of the host plants (cocoa, kolanut and bush mango) with only slight differences with respect to individual host plants. The results of the mineral analysis of the major cations further proves that *Phragmanthera incana* is not host specific having major cations like potassium, magnesium, calcium and sodium in varying quantities on the three plant hosts (cocoa, kolanut and bush mango) and their respective mistletoe.
